# Food selection associated with sense of coherence in adults

**DOI:** 10.1186/1475-2891-4-9

**Published:** 2005-02-28

**Authors:** Ulrika Lindmark, Birgitta Stegmayr, Berit Nilsson, Bernt Lindahl, Ingegerd Johansson

**Affiliations:** 1 Department of Natural Sciences and Biomedicine, University of Jönköping, Jönköping, Sweden; 2 Department of Public Health and Clinical Medicine, Umeå university, Umeå, Sweden; 3 Department of Odontology, Umeå university, Umeå, Sweden

**Keywords:** Diet, Sense of coherence, Food frequency questionnaire, Northern Sweden MONICA study

## Abstract

**Background:**

Favorable dietary habits promote health, whereas unfavorable habits link to various chronic diseases. An individual's "*sense of coherence" *(SOC) is reported to correlate with prevalence of some diseases to which dietary habits are linked. However, understanding what determines an individual's dietary preferences and how to change his/her behavior remains limited. The aim of the present study was to evaluate associations between dietary intake and SOC in adults.

**Methods:**

Diet intake was recorded by an 84-item semi-quantitative food frequency questionnaire and SOC was measured by the 13-item Antonovsky questionnaire in 2,446 men and 2,545 women (25–74 years old) from the population based northern Sweden MONICA screening in 1999.

**Results:**

Intakes of energy, total and saturated fat, ascorbic acid, sucrose, and servings of fruits, vegetables, cereals, and sweets correlated with SOC among women, whereas intakes of total and saturated fat, ascorbic acid, fiber, and alcohol, and servings of fruits, vegetables, bread, bread and cereals, fish, and potatoes correlated with SOC among men. With a few exceptions, intakes of these nutrients/foods were significantly explained by SOC quartile scores in linear GLM models. Both women and men classified into the highest SOC quartile had significantly higher age-BMI-education standardized mean intakes of vegetables than those in the lowest quartiles. Women in the highest SOC quartile also had higher intake of fruits but lower intakes of energy, total and saturated fat, sucrose, and sweets.

Projection to latent structures (PLS) multivariate modeling of intakes of the 84 food items and food aggregates simultaneously on SOC scores supported low SOC to coincide with a presumably less health promoting dietary preference, e.g. intake of pizza, soft drinks, candies, sausages for main course, hamburgers, mashed potato, chips and other snacks, potato salad, French fries, whereas men and women with high SOC scores were characterized by e.g. high intake of rye crisp whole meal bread, boiled potato, vegetables, berries, and fruits.

**Conclusion:**

Both men and women in the highest, as compared with the lowest, SOC score quartile reported more "healthy" food choices. Dietary habits for individuals in the lowest SOC quartile therefore may render a higher risk for various endemic diseases.

## Background

Favorable dietary habits promote health, whereas unfavorable habits are linked to development of various chronic diseases, such as cardiovascular diseases, type-2 diabetes and dental caries. An excess intake of energy, excess refined sugars and saturated fatty acids, and too little fibers and antioxidants can contribute to the development of chronic diseases [1-4]. In general a favorable dietary pattern is characterized by a rich content of fruits, vegetables and fiber-rich cereal products and a low content of fat and refined sugar. A diet rich in saturated fat and sugar, but low in fiber, fruit and vegetables is considered unfavorable [5]. The public health message during the latest decades, i.e. to reduce total fat intake, especially saturated fat, and to eat more vegetables, fruits and whole meal cereals, has been constant [6,7].

An individual's dietary pattern is largely set by cultural traditions and availability, but both physiological and psychological influences have been described [8]. However, understanding what determines an individual's dietary preferences and how to change his/her behavior is limited. The medical sociologist, Aaron Antonovsky [9] was of the opinion that approaches to health and disease can be either salutogenic (origins of health) or pathogenic (origins of disease). He showed health to be connected to an individual's "*Sense of coherence" *(SOC), and "*generalized resistance resources*" (GRRs), such as income, education, ego strength, knowledge, which would provide energy to combat various stressors, and thus influential factors in the salutogenic model. Central to sense of coherence are comprehensibility (the cognitive component), manageability (the instrumental component), and meaningfulness (the emotional component). Individuals with low SOC scores are reported to have a higher frequency of various diseases [10-13], some of which are also linked to dietary habits [14-17]. An association between SOC and food selection/eating pattern is indicated by the reported (*i*) higher sucrose intake in adolescents with low SOC scores [14], (*ii*) lower ability to change dietary habits and lose weight in over-weight individuals with moderate to low SOC scores [15], and (*iii*) better blood sugar control in type-2 diabetics with high SOC scores, whereas those with low scores have poorer sugar balance [16].

The aim of the present study was to evaluate the association between dietary intake and SOC in adults. The hypothesis was that low SOC scores were associated with less favorable habits and vice versa.

## Methods

### Study population

The Northern Sweden MONICA Project was performed in Västerbotten and Norrbotten, the two most northerly counties in Sweden, with a total population of around half a million inhabitants and a high prevalence of cardiovascular disease. Surveys were performed in 1986, 1990, 1994 and 1999 [18]. In each of the age groups 25–34, 35–44, 45–54 and 55–64 years 250 men and 250 women were randomly selected and invited to participate. In 1994 and 1999, the age group 65–74 years was added. Every person selected was invited to an examination at the nearest health care centre.

In the 1999 survey, all participants from the three previous cohorts (n = 5,129), as well as a new cross-sectional sample of 2,500 randomly selected people, were invited. In total 6,000 individuals (71.8 %) participated. For the present paper all individuals aged 75 years or older (n = 206) were excluded.

In the MONICA project various cardiovascular risk factors, including weight, height and education level, were monitored. Education was grouped into three levels: primary school (≤ 9 years), secondary school (10–12 years) and university education (≥ 13 years). Body mass index (BMI) was calculated as the ratio between weight (kg) and height^2 ^(m). BMI was grouped as <20 underweight, 20–24.9 normal weight, 25 – 26.9 moderate overweight, 27 – 29.9 overweight, and ≥ 30 obese. Special forms for questions about food intake and sense of coherence were added.

### Recording of sense of coherence (SOC)

Sense of coherence was monitored using the 13-item questionnaire by Antonovsky [9,19]. Participants who had not answered all 13 questions were excluded. The SOC scores, with a theoretical range from 13 to 91, were calculated as described previously [9,10,19]. High scores denote strong SOC.

### Recording of dietary intake

The subjects were requested to complete a self-administered, semi-quantitative and optically readable food frequency questionnaire [20]. Frequencies of consumption of 84 food items were reported on an increasing, nine-level scale, including never, maximum once a month, 1–3 times per month, once a week, 2–3 times a week, 4–6 times a week, once a day, 2–3 times a day, and 4 or more times a day. The questionnaire included eight questions on various types of fats, nine on milk and other dairy products, eight on bread and cereals, ten on fruit, greens and root vegetables, and nine on soft drinks and sugar-containing snacks, and five questions on spirits, wine and beer. Twenty nine of the remaining 35 questions recorded intake of potatoes, rice, pasta, meat and fish, and six varied items, such as salty snacks, coffee, tea and juice. The respondents indicated their average portion of a) potatoes/pasta/rice, b)vegetables and c) meat/ground meat/sausages/fish by comparison of four color photos illustrating four plates with increasing portion sizes of potato, vegetables and meat. For the other food items, we assumed gender- and age-standard portion sizes [20].

The reported frequencies of consumption were converted to number of intakes per day, and energy and nutrient intakes were calculated by multiplying these frequencies by portion size and energy or nutrient content from a food composition database from the Swedish National Food Administration. The energy and nutrient contents were calculated using the software MAT's (Rudans Lättdata, Sweden).

Participants who had more than 10% missing answers were excluded. Single missing answers in sections where normally only one of several options is consumed frequently, such as type of milk, were not grounds for exclusion. Nutrient intake could not be estimated if portion size indication was missing.

### Statistical methods

Data were analyzed separately for men and women using the SAS System for Windows (Release 8.02, SAS Institute, Cary, NC). Dietary variables were logarithmically transformed to improve normality. Univariate Pearson correlation coefficients were calculated between ln-transformed diet measures and SOC score. Differences between means for men and women were tested with Student's *t-*tests, and differences between more groups, i.e., age, BMI and education level groups, by ANOVA. P-values ≤ 0.05 were considered significant. Dietary intake and reporting thereof are highly influenced by gender, age, BMI, and educational level [21]. Therefore, mean intakes were standardized for age, BMI and educational level using general linear model (proc GLM). Significance of food item/nutrient predictors was based on (*i*) type I and (*ii*) type-III sum of square estimates, where (*i*) corresponds to a univariate regression, i.e., only the SOC score had entered the model, and (*ii*) corresponds to a multiple regression, i.e., all independent variables (SOC, gender, age, BMI, and educational level) were included in the model.

To evaluate food selection pattern in individuals in relation to SOC score, PLS multivariate projection to latent structures was applied [22]. PLS is a method to relate two data matrices X and Y to each other by linear multivariate modeling. In contrast to traditional linear modeling, co-varying variables may be included. The PLS parameters carrying information about the x- and y- variables, e.g. R^2^, Q^2 ^and VIP, were generated as previously described [22]. The R^2^- and Q^2^-values give the capacity of the X matrix to explain (R^2^) and predict (Q^2^; equals cross-validated R^2^) the variance of the Y matrix. The relative importance of each x-variable for the correlation structure among X and Y is given as a VIP-value (Variables of Importance in the Projection); VIP-values >1.0 are influential and VIP-values ≥ 1.5 highly influential.

## Results

In total 2,446 men and 2,545 women answered the SOC- and food-frequency-questionnaires in such a way that the inclusion criteria were met. This corresponded to 86.6% of the 25–74 year old participants in the northern Sweden MONICA 1999 study.

**Figure 1:**
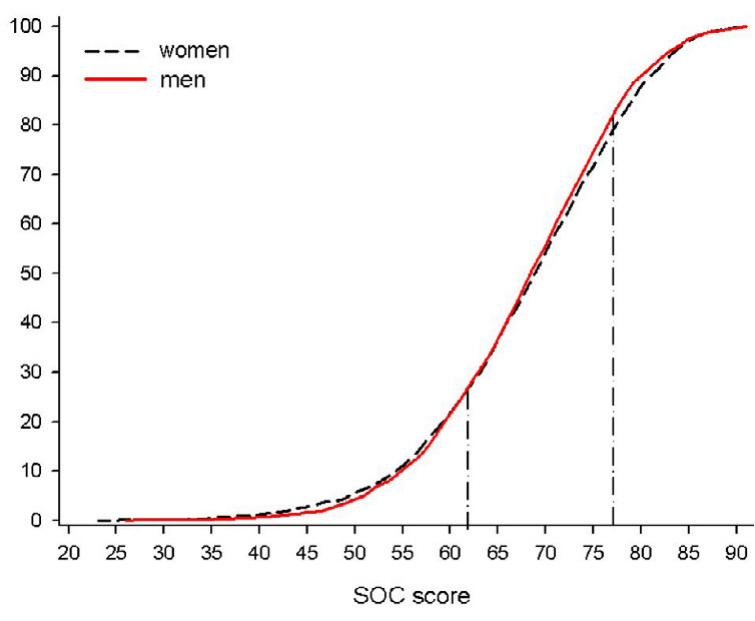
Cumulative frequencies of SOC scores in men and women. The vertical lines denote the upper and lower cut-off values for the lowest and highest quartiles, respectively.

In the present cohort SOC scores varied from 23 to 91 points (Fig. 1). Means in quartile groups based on SOC distribution increased by approximately 9 points per quartile among both men and women (see Additional file 1). Mean scores neither differed significantly between men and women, nor between normal weight, overweight or obese individuals, nor between groups with different length of education. However, mean scores increased significantly with increasing age in both sexes (see Additional file 1).

Univariate correlation analyses between SOC scores and daily intake of nutrients or servings per week in food groups revealed significant associations among women for intakes of energy (kCal/day), total and saturated fat (g/week), ascorbic acid (mg/week), sucrose (g/week), and servings/week of fruits, vegetables, cereals, and sweets (data not shown). Among men significant correlations were seen for daily intake of total and saturated fat (g/day), ascorbic acid (mg/day), fiber (g/day), and alcohol (g/day), and servings of fruits, vegetables, bread, bread and cereals, fish, and potatoes (data not shown). Except for ascorbic acid and cereal intake in women, and bread, bread and cereals, and potato intake in men, variations in intakes of these foods/nutrients were significantly explained by SOC quartile scores in linear GLM models (Type I SS; Tables 1 and 2). After standardization for age, BMI and education level SOC quartile scores still contributed in explaining intake of saturated fat, vegetables, sucrose and sweets in women and vegetables and alcohol in men (Type III SS; Tables 1 and 2). In accordance, mean (age, BMI and education standardized) intakes of vegetables were significantly higher in women and men (1.1 and 0.8 servings more per week, respectively) in the highest, compared to the lowest, quartiles (Tables 2 and 3). Women in the highest quartile also had higher intakes of fruits (+1.0 serving/week), but lower intake of energy (-365 kCal/week), total and saturated fat (-15 and -8 g/week, respectively), sucrose (-14 g/week), and sweets (-1.3 servings/week) (Table 1).

PLS multivariate modeling of intakes of the 84 single food items and food aggregates simultaneously as an X-matrix on SOC-scores (Y-matrix) rendered models which supported low SOC to coincide with a less health promoting dietary preference and vice versa. Thus, in both men and women low SOC scores coincided with high intake of pizza, soft drinks, candies, sausages for main course, hamburgers, mashed potato, chips and other snacks, potato salad, French fries, and traditional broth soaked bread and potato dumplings (Table 3, items in bold). In contrast, both men and women with high SOC scores were characterized by a high intake of rye crisp bread (whole meal), boiled potato, and vegetables (Table 3, items in bold). In addition, some more gender specific characteristic food selections are seen in Table 3.

## Discussion

The present study shows that a high sense of coherence (SOC) is associated with health-promoting food choices, whereas low scores are associated with a less favorable food pattern. SOC score independently contributed in explaining variations in intakes of vegetables in both men and women, and in intakes of saturated fat, sucrose, sweets, and fruits among women, and alcohol intake among men. Correlations for other nutrient/food intakes seen in the initial univariate evaluations, disappeared when tested together with age, BMI and educational level in multiple linear GLM modeling. This indicates that some dietary preferences and food selections are linked to socioeconomic status, whereas others are more bound to the individual's sense of coherence, i.e. his/her personal way of grasping and handling life situations.

Although the present results support earlier findings that associate high SOC scores with a more healthy diet [14-17], the interpretations should be considered in relation to methodological strengths and limitations of the study. The Northern Sweden MONICA study cohort of 1999 consisted of 6,000 participants, corresponding to 71.8 % of the invited subjects. Of these 86% could be included in the present evaluation. All subjects were primarily invited to the study by random from a continuously updated population ledger. Taken together, this allows for generalization of the results to a population level, at least to a population similar to that in northern Sweden. Within the MONICA project, extensive quality assessments of various methods and measures have been performed [23], and both the SOC and food frequency questionnaires were printed in an optically readable format to minimize errors due to faulty entry of data. Furthermore, the food frequency questionnaire has been found valid in a random sub-sample of representative adults [20], and the Cronbach alpha value [24] among SOC answers, was 0.81, which is in accordance with other studies [10,13,15], indicates reliability and internal consistency among the answers [19]. However, the cross-sectional design of the study, where the SOC scores were measured at the same time as the food preferences, precludes the possibility of interpreting the identified associations as causally related.

The sense of coherence instrument has been constructed to measure an individual's capacity to cope in a salutogenic way [9]. In accordance with this the present study shows that the SOC level is associated with a "healthy" eating pattern. It has previously been shown that physically active individuals have higher SOC scores than inactive persons [25,26]. The hypothesis that SOC links to life style in a wider sense is therefore supported, even though we are aware that it cannot be ruled out that a healthy life style *per se *may influence SOC. The four cross-sectional MONICA screenings in 1986, 1990, 1994 and 1999 have demonstrated a distinct time trend in dietary intake in northern Sweden [27]. The present results therefore raise some questions. Are individuals with higher SOC more prone to change eating pattern? Are those changes more likely to follow dietary recommendations? Is a low SOC level a marker for a less healthy life style in general? Since all previous participants were re-called in 1999 answers may be searched for within the frame work of the MONICA project.

Notably, both men and women in the highest, as compared with the lowest SOC score quartile, reported more food choices from the contemporary public message of a healthy life style [6,7]. In contrast, food selection in those in the lowest SOC-score quartile may render these individuals at a higher risk for various chronic diseases. Further knowledge of the influence of an individual's sense of coherence on his/her willingness and ability to change dietary habits may be of advantage when designing preventive programs.

## Conclusion

Both men and women in the highest, as compared with the lowest, SOC score quartile reported more "healthy" food choices. Dietary habits for individuals in the lowest SOC quartile therefore may render a higher risk for various endemic diseases.

## Competing interests

The author(s) declare that they have no competing interests

## Authors' contributions

All participants have contributed to study design and manuscript preparation; IJ has been main author, UL has done data processing, BN has been responsible for SOC, BL for general behavior aspects, and BS is PI for the Northern Sweden MONICA study.

## Supplementary Material

Additional File 1 SOC scores in gender, age, BMI, education level and SOC-quartile groups. Differences between means for men and women are tested with t-test, and among more groups, i.e. age groups, BMI education level, with ANOVA. ns for p > 0.05 Click here for file

## Figures and Tables

**Table 1:** Diet intake in women. Food items with significant univariate correlations between diet intake and SOC score are listed. ns for >0.05.

	**GLM PROCEDURE^1^**		**MEAN INTAKES OF NUTRIENTS AND FOODS^4^**			
		
**NUTRIENT/FOOD ITEM**	**TYPE I^2^**	**TYPE III^3^**	**Q1 (LOW)**	**Q2+Q3**	**Q3 (HIGH)**	**Q1 VS Q4^5^**
Energy (kCal/day)	0.066	ns^a,b,e^	1 531	1 492	1 478	0.037
Fat total (g/week)	0.012	ns^a,b^	373	362	358	0.027
Fat saturated (g/week)	0.017	ns^a,b^	154	148	146	0.023
Ascorbic acid (mg/week)	ns	ns^a,e^	520	528	533	ns
Fruits (servings/week)	0.028	0.088^a,e^	8.0	8.5	8.9	0.018
Vegetables (servings/week)	0.007	0.017^a,e^	8.6	9.2	9.7	0.002
Cereals (servings/week	ns	ns^a,b^	3.1	3.1	3.5	ns
Sucrose (g/week)	0.037	0.067^a,b^	188	181	174	0.011
Sweets (servings/week)	0.014	0.019^a,b^	8.8	8.1	7.5	0.002

1) GLM modeling of SOC-quartile classification scores, including age, BMI, and level of education group in the model, ln-transformed diet intake values. ns for p > 0.09.

2) Type I sum of square estimates are when only SOC quartile scores have entered the model, and

3) Type III sum of square estimates when all variables are kept in the model. Independent contribution by (a) age group, (b) BMI, and (e) education level in explaining nutrient/food intake variations are indicated by the respective superscript.

4) Geometric mean intakes standardized for age, BMI, and education in SOC-quartile (Q1, Q2+Q3, Q4) groups. Mean (95% CI) SOC score in the Q2+Q3 group (n_women _= 1303, 49.6% of all women) is 69.2 (69.0–69.4).

5) The difference between mean intakes (standardized for age, BMI and education) in the lowest (Q1) versus highest (Q4) SOC quartile groups.

**Table 2:** Diet intake in men. Food items with significant univariate correlations between diet intake and SOC score are listed. ns for >0.05.

	**GLM PROCEDURE^1^**		**MEAN INTAKES OF NUTRIENTS AND FOODS^4^**			
		
**NUTRIENT/FOOD ITEM**	**TYPE I^2^**	**TYPE III^3^**	**Q1 (LOW)**	**Q2+Q3**	**Q3 (HIGH)**	**Q1 VS Q4^5^**
Energy (kCal/day)	ns	ns^a,b^	1 964	1 980	1 936	ns
Fat total (g/week)	0.001	0.020^a,b,e^	545	545	525	0.064
Fat saturated (g/week)	0.006	0.057^a,b,e^	227	227	219	ns
Ascorbic acid (mg/week)	0.021	ns^a,e^	428	456	458	0.063
Fiber (g/week)	0.006	ns^a,b^	123	127	127	ns
Fruits (servings/week)	0.058	ns^a,e^	5.2	5.6	5.5	ns
Vegetables (servings/week)	0.009	0.037^a,e^	5.6	6.2	6.4	0.009
Bread (servings/week)	ns	ns^a,b,e^	17.0	17.6	17.4	ns
Bread and cereals (servings/week)	0.039	ns^a,b,e^	21.5	22.3	22.2	ns
Fish (servings/week)	0.010	ns^a,e^	1.0	1.1	1.1	ns
Potato (servings/week)	ns	ns^a^	4.9	5.0	4.9	ns
Alcohol (g/week)	0.007	0.011^a,b,e^	16.1	23.1	20.5	0.064

1) GLM modeling of SOC-quartile classification scores, including age, BMI, and level of education group in the model, ln-transformed diet intake values. ns for p > 0.09.

2) Type I sum of square estimates are when only SOC quartile scores have entered the model, and

3) Type III sum of square estimates when all variables are kept in the model. Independent contribution by (a) age group, (b) BMI, and (e) education level in explaining nutrient/food intake variations are indicated by the respective superscript.

4) Geometric mean intakes standardized for age, BMI, and education in SOC-quartile (Q1, Q2+Q3, Q4) groups. Mean (95% CI) SOC score in the Q2+Q3 group (n_men _= 1323; 50.4% of all men) is 69.3 (69.0–69.5).

5) The difference between mean intakes (standardized for age, BMI and education) in the lowest (Q1) versus highest (Q4) SOC quartile groups.

**Table 3:** Food selection pattern by SOC score. VIP-values denotes the influence in explaining variation in SOC-scores. VIP values ≥ 1.2 are listed, and + denotes associations with high SOC and – association with low SOC scores.

**MEN**			**WOMEN**		
	
FOOD ITEM	ASSOCIATION	VIP	FOOD ITEM	ASSOCIATION	VIP
***Influential for high SOC***			***Influential for high SOC***		
Bread	+	2.18	Fruits	+	1.83
Bread and cereals	+	1.96	**Vegetables**	+	1.83
**Rye crisp bread (whole meal)**	+	1.89	Liver paste	+	1.64
Fish	+	1.76	**Boiled potato**	+	1.47
**Boiled potato**	+	1.71	**Rye crisp bread (whole meal)**	+	1.43
Berries	+	1.51	Blood foods	+	1.32
Wine	+	1.49	Cereals	+	1.29
Light beer	+	1.32	Oat flake porridge	+	1.24
**Vegetables**	+	1.23			
					
***Influential for low SOC***			***Influential for low SOC***		
**Pizza**	-	2.41	**Mashed potato**	-	2.98
**Soft drinks**	-	2.25	**Sausage as main course**	-	2.11
Pasta	-	2.14	**Potato salad**	-	1.98
**Candies**	-	2.02	**Soft drinks**	-	1.94
**Sausage as main course**	-	1.97	**French fries**	-	1.89
**Hamburgers**	-	1.94	**Hamburgers**	-	1.82
**Mashed potato**	-	1.87	Beer (high alcohol)	-	1.71
Fried potato	-	1.65	**Pizza**	-	1.68
Butter for cooking	-	1.63	Sweets	-	1.67
**Chips, cheese doodles, nuts**	-	1.57	**Candies**	-	1.60
**Potato salad**	-	1.48	Rice	-	1.50
Banana	-	1.37	Pancakes	-	1.43
**French fries**	-	1.22	Syrups	-	1.42
**Traditional broth soaked bread**	-	1.21	Cookies	-	1.39
Fruit soup	-	1.21	**Traditional broth soaked bread**	-	1.29
Traditional potato dumpling	-	1.20	**Chips, cheese doodles, nuts**	-	1.29
