# Supplementary material for: Food selection associated with sense of coherence in adults
Source: Nutr J. 2005 Feb 28;4:9. doi: 10.1186/1475-2891-4-9 (PMC554973; doi:10.1186/1475-2891-4-9)
Supplement: Additional File 1 — SOC scores in gender, age, BMI, education level and SOC-quartile groups. Differences between means for men and women are tested with t-test, and among more groups, i.e. age groups, BMI education level, with ANOVA. ns for p > 0.05 [file 1475-2891-4-9-S1.pdf]

		SOC score				P-Value		
		Women		Men		T-test	ANOVA among groups	
		N	mean (95% CI)	N	mean (95% CI)	between sexes	women	men
Gender		2 545	68.5 (68.1-68.9)	2 446	68.4 (68.0-68.8)	ns	-	-
Age	25-34 yrs	217	67.6 (66.0-69.1)	189	65.5 (64.1-67.0)	ns	0.039	0.0001
	35-44 yrs	512	67.6 (66.7-68.5)	450	66.4 (65.5-67.4)	ns		
	45-54 yrs	641	68.6 (67.7-69.4)	653	68.3 (67.6-69.1)	ns		
	55-64 yrs	659	68.7 (67.9-69.5)	619	69.7 (68.9-70.4)	ns		
	65-74 yrs	516	69.5 (68.6-70.4)	535	69.6 (68.7-70.4)	ns		
BMI	<25	1 131	68.4 (67.8-69.0)	775	68.2 (67.5-68.9)	ns	ns	ns
	≥25-<27	440	69.2 (68.4-70.1)	609	68.6 (67.9-69.4)	ns		
	≥27-<30	489	68.6 (67.6-69.5)	665	68.6 (67.8-69.3)	ns		
	≥30	478	68.2 (67.2-69.1)	397	67.9 (66.9-68.9)	ns		
Education	≤9 yrs	870	68.9 (68.2-69.6)	967	68.8 (68.2-69.4)	ns		
	≥10-≤12 yrs	1 007	68.1 (67.4-68.7)	997	68.0 (67.4-68.6)	ns		
	≥13 yrs	668	68.6 (67.8-69.4)	482	68.2 (67.3-69.2)	ns		
SOC quartile	Q1 (low)	618	54.2 (53.7-54.7)	588	55.0 (54.6-55.5)	0.035	ns	ns
	Q2	663	65.7 (65.5-65.9)	689	65.8 (65.6-65.9)	ns		
	Q3	640	72.9 (72.8-73.1)	634	73.0 (72.8-73.1)	ns		
	Q4 (high)	624	81.2 (80.9-81.4)	535	80.9 (80.6-81.2)	ns		
